# 341. Attitudes and Experiences of Individuals Approached to Participate in a Randomised, Placebo-Controlled, Feasibility Study of Encapsulated Faecal Microbiota Transplantation (FMT) for the Eradication of Gastrointestinal (GI) Carriage of Antibiotic Resistant Organisms (ARO): a Nested Qualitative Study

**DOI:** 10.1093/ofid/ofad500.412

**Published:** 2023-11-27

**Authors:** Blair Merrick, Désirée Prossomariti, Michael Kertanegara, David Wyatt, Simon D Goldenberg

**Affiliations:** Guy's and St Thomas' NHS Foundation Trust and King's College, London, London, England, United Kingdom; Guy's and St Thomas' NHS Foundation Trust, London, England, United Kingdom; Guy's and St Thomas' NHS Foundation Trust, London, England, United Kingdom; King's College, London, London, England, United Kingdom; Guy's and St Thomas' NHS Foundation Trust and King's College, London, London, England, United Kingdom

## Abstract

**Background:**

We undertook a single-centre, randomised placebo-controlled study to assess feasibility of eradicating GI carriage of ARO using encapsulated, lyophilised FMT – FERARO – IRCTN reg. no. 34467677. To aid design of future substantive trial, a nested qualitative study collected participant experiences and perceptions.

**Methods:**

A subset (n=24) of recruited participants were approached and 13/24 (54%) participated in one of two 2-hour in-person semi-structured focus groups during, or shortly after completion of, their 6-month study follow-up. Additionally, where given, reasons for non-participation in approached eligible individuals were collected (n=110). Qualitative data underwent thematic analysis; initial coding and theme identification were undertaken by 2 trial researchers independently before further analysis in collaboration with third independent qualitative researcher.

**Results:**

Four central themes were identified; (i) barriers to, and (ii) drivers of, trial participation, (iii) participant randomised controlled trial experience, and (iv) support of future research projects. Participation was influenced by physical ability and time availability to complete study related activities, prior treatment journey and experiences, as well as ongoing available treatment options, and altruism - a willingness to give back to the National Health Service (NHS). Importantly, neither the Covid-19 pandemic, nor lack of acceptability (‘yuck’ factor) of encapsulated lyophilised FMT, were identified as barriers to participant recruitment. FMT was well tolerated, and an intervention that participants viewed as ‘natural’ when compared to other medical treatments. Participants reported that both physical and mental health and/ or health behaviours improved as a result of trial participation, regardless of treatment allocation.

**Figure:**
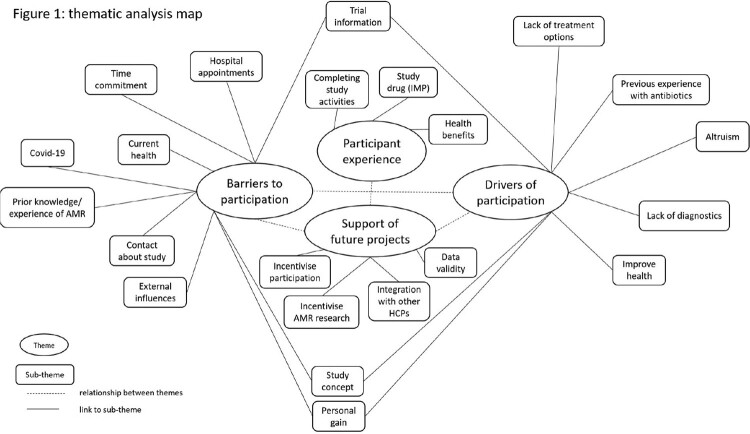

Themes, subthemes, and their relationships.

**Conclusion:**

The frequency of face-to-face hospital-based study appointments presented a barrier to study participation, particularly for less mobile, more comorbid patients – a cohort that may benefit the greatest from participation. Substantive trial recruitment may be incentivised by the use of remote and/ or home visits including for investigational medicinal product (IMP) administration.

**Disclosures:**

**Simon D. Goldenberg, FRCPath, MD (Res), PGDipID, DipHIC, MSc**, EnteroBiotix: Honoraria|Tillotts Pharma UK: Honoraria

